# Online Video Teletherapy Treatment of Obsessive-Compulsive Disorder Using Exposure and Response Prevention: Clinical Outcomes From a Retrospective Longitudinal Observational Study

**DOI:** 10.2196/36431

**Published:** 2022-05-19

**Authors:** Jamie D Feusner, Nicholas R Farrell, Jeremy Kreyling, Patrick B McGrath, Andreas Rhode, Ted Faneuff, Stephanie Lonsway, Reza Mohideen, John E Jurich, Larry Trusky, Stephen M Smith

**Affiliations:** 1 NOCD Inc Chicago, IL United States; 2 Department of Psychiatry University of Toronto Toronto, ON Canada; 3 General Adult Psychiatry & Health Systems Division Centre for Addiction and Mental Health Toronto, ON Canada; 4 Department of Women's and Children's Health Karolinska Institutet Stockholm Sweden

**Keywords:** digital behavioral health, cognitive-behavioral therapy, CBT, exposure and ritual prevention, ERP, EX/RP, digital health, telehealth, cognitive therapy, obsessive compulsive disorder, OCD, clinical outcomes, teletherapy, remote therapy, telemedicine, obsessive compulsive, symptom, comorbid symptom, comorbidity, comorbidities, video therapy, virtual therapy, clinical outcome, patient outcome, online therapy, mobile health, mHealth, app based, health app, technology assisted, messaging

## Abstract

**Background:**

Exposure and response prevention, a type of cognitive-behavioral therapy, is an effective first-line treatment for obsessive-compulsive disorder (OCD). Despite extensive evidence of the efficacy of exposure and response prevention (ERP) from clinical studies and in real-world samples, it is still underused as a treatment. This is likely due to the limits to access to care that include the availability of adequately trained therapists, as well as geographical location, time, and cost barriers. To address these, NOCD created a digital behavioral health treatment for OCD using ERP delivered via video teletherapy and with technology-assisted elements including app-based therapy tools and between-session therapist messaging.

**Objective:**

We examined treatment outcomes in a large naturalistic sample of 3552 adults with a primary OCD diagnosis who received NOCD treatment.

**Methods:**

The treatment model consisted of twice-weekly, live, face-to-face video teletherapy ERP for 3 weeks, followed by 6 weeks of once-weekly brief video teletherapy check-ins for 30 minutes. Assessments were conducted at baseline, at midpoint after completion of 3 weeks of twice-weekly sessions, and at the end of 6 weeks of brief check-ins (endpoint). Longitudinal assessments were also obtained at 3, 6, 9, and 12 months after endpoint.

**Results:**

Treatment resulted in clinically and statistically significant improvements, with a 43.4% mean reduction in obsessive-compulsive symptoms (g=1.0; 95% CI 0.93 to 1.03) and a 62.9% response rate. Treatment also resulted in a 44.2% mean reduction in depression, a 47.8% mean reduction in anxiety, and a 37.3% mean reduction in stress symptoms. Quality of life improved by a mean of 22.7%. Reduction in OCD symptoms and response rates were similar for those with mild, moderate, or severe symptoms. The mean duration of treatment was 11.5 (SD 4.0) weeks, and the mean total therapist time was 10.6 (SD 1.1) hours. Improvements were maintained at 3, 6, 9, and 12 months.

**Conclusions:**

In this sample, representing the largest reported treated cohort of patients with OCD to date, video teletherapy treatment demonstrated effectiveness in reducing obsessive-compulsive and comorbid symptoms and improved quality of life. Further, it achieved meaningful results in less than half the total therapist time compared with standard once-weekly outpatient treatment, an efficiency that represents substantial monetary and time savings. The effect size was large and similar to studies of in-person ERP. This technology-assisted remote treatment is readily accessible for patients, offering an advancement in the field in the dissemination of effective evidence-based care for OCD.

## Introduction

Obsessive-compulsive disorder (OCD) is a prevalent and disabling psychiatric disorder, affecting 2.3% of individuals during their lifetimes [1]. Typically chronic if untreated, OCD is markedly detrimental to one’s quality of life [2]. Yet, OCD can be treated effectively with psychotherapy or pharmacological interventions [3]. Exposure and response prevention (ERP), also known as exposure and ritual prevention, is a type of cognitive-behavioral therapy (CBT) that consistently demonstrates efficacy for OCD in numerous controlled trials and is also effective in less controlled clinical settings [3-7]. Based on this research evidence, ERP is considered a first-line treatment for OCD [8,9].

However, ERP requires specialty-trained therapists and thus is not readily available to everyone with OCD because of limited numbers of trained therapists, as well as cost and geographical limitations [10]. Indeed, the majority of individuals with OCD and related anxiety conditions are unable to access evidence-based psychotherapy [11]. Moreover, ERP typically requires over 25 hours of therapist time per patient [12] to achieve meaningful results; thus, when delivered in its most common format of once-weekly outpatient therapy, it could take 6 or more months.

To address the challenges of delivering ERP in terms of barriers to access and associated cost and time, NOCD has developed a digital behavioral health treatment program using video teletherapy. Remote ERP for OCD, delivered by video or telephone, has been demonstrated to significantly improve OCD symptoms [13]. Two head-to-head comparisons with in-person treatment in adults and adolescents show only small differences in outcome [14,15]. One of the several vital advantages of remote treatment is that therapists can readily interact with patients in the specific settings that most trigger their obsessional thoughts, images, or urges, for example in the home. This allows for administering in-session exposures that otherwise could be difficult or impossible to reproduce in an office setting. Although therapists in traditional face-to-face treatments can visit patients’ homes and other nonoffice settings to administer exposures and help patients practice response prevention, this is logistically challenging and inefficient due to the travel times involved. Moreover, as of 2022, approximately 83% of the world’s population (6.5 billion) owns a smartphone [16], and this grows yearly.

NOCD’s treatment approach was inspired by a treatment previously tested in an open clinical trial [17] (N=33) that used the NOCD app integrated with brief in-person therapy. This trial tested a treatment protocol designed to minimize therapist time while increasing therapy intensity compared with once-weekly ERP sessions. It is possible that greater symptom reduction earlier in treatment, which may occur with more intensive treatment, could portend better ultimate clinical outcomes [18-20]. In a 2020 trial conducted by Gershkovich et al [17], there were high satisfaction ratings: 68.2% were “very” and 31.8% “mostly” satisfied with the services received. The treatment resulted in a mean reduction in OCD symptoms of 38.9%, with a response rate (≥35% reduction in OCD symptoms) of 52%. Mean therapist time was 6.7 (SD 1.52) hours total per patient.

We designed a treatment model for NOCD to treat patients with OCD using exposure and response prevention, with similar intensity, and to be able to reach as many as possible in the general community. To provide accessibility, all sessions were conducted remotely with video teletherapy. To provide additional support, enhance adherence, and potentially improve efficacy, every patient had access to between-session contact with their therapist via messaging. Further, a large online OCD community was available for further support through group message boards and scheduled support group sessions. In addition, peer support from individuals who had completed NOCD treatment was available to patients prior to starting treatment. The objective of this study was to examine treatment outcomes in a large naturalistic sample of 3552 adults with a primary OCD diagnosis who received NOCD treatment from January 1, 2020, to June 30, 2021.

## Methods

### Diagnostic Evaluations and Inclusion and Exclusion Criteria

Patients initially contacted the NOCD intake team as self-referrals or as referred from their health plans. They underwent diagnostic assessments by licensed clinical psychotherapists, who had received standardized training from NOCD in the evaluation and treatment of OCD using ERP. The diagnostic assessment consisted of a comprehensive clinical evaluation, including biopsychosocial elements of their history, and a standardized, semistructured diagnostic evaluation using the Diagnostic Interview for Anxiety, Mood, and Obsessive Compulsive and Related Neuropsychiatric Disorders (DIAMOND) [21]. Individuals who met DIAMOND criteria for OCD (consistent with Diagnostic and Statistical Manual of Mental Disorders, Fifth Edition [22]) as their primary disorder were treated. The majority of those who scored a 7 (“extreme”) on the DIAMOND clinician-rated severity scale were referred to higher levels of care, including intensive outpatient programs, partial hospitalization programs, or residential treatment programs (exceptions were made on a case-by-case basis for a small number of individuals [n=16] whom the therapist and clinical leadership deemed may benefit from treatment by NOCD). Other situations that resulted in referral at the time of diagnostic assessment included active substance use disorders or comorbid uncontrolled psychiatric disorders or symptoms (eg, mania, psychosis, or active suicidality), deemed to potentially interfere with treatment, on a case-by-case basis. Although the current analysis is of patients 18 years and older, NOCD treated those 5 years of age and older (results from the child and adolescent cohort are forthcoming). There was no upper limit on age. Medicated or unmedicated individuals were treated.

### Treatment Model

The NOCD treatment model consisted of twice-weekly 60-minute remote ERP video sessions for 3 weeks. After this, patients had 6 weeks of once-weekly 30-minute video “check-in” sessions to guide ongoing ERP homework assignments conducted by the patients.

Therapists were trained and instructed to follow this framework for treatment but were allowed some flexibility to add sessions, if needed. In addition, between sessions, all patients had access to as-needed asynchronous text messaging with their therapists 5 out of 7 days per week to obtain guidance with exposures and response prevention. Patients had 24 hours per day and 7 days per week access to the online NOCD community, consisting of a forum of individuals around the world self-identified as having OCD, providing support and advice through online (monitored) postings. The NOCD app was available for patients to use during treatment; it provided tools for patients, in collaboration with their therapists, to create exposure hierarchies and do exposure exercises. Patients could also read and post messages in the NOCD community through the app.

All sessions were conducted via Zoom (US Health Insurance Portability and Accountability Act–compliant version). Patients could join the sessions via any personal computer or portable electronic device. For billing purposes, both the therapist and patient needed to be on video throughout the session. Aside from their electronic device, there was no other hardware required for either patients or therapists. Therapists were trained to not proceed with sessions if adequate sound and video quality could not be achieved, and in these scenarios, to reschedule in a timely manner. Additionally, during traditional daytime business hours (when most sessions were held) there was live technical support available to therapists to assist patients with troubleshooting if there were connectivity issues.

Therapists had Master’s, PhD, or PsyD degrees, and were licensed in the states in which they provided remote treatment. Therapists received training by NOCD to conduct ERP and were provided ongoing group and individual supervision by experienced NOCD clinical leadership team members. All NOCD therapists received 3 days of intensive training on OCD, ERP, and application of ERP to OCD. After this training, there are several assessments that all clinicians must pass, including quizzes, a mock diagnostic session, a mock education session, and mock ERP sessions. As therapists go live, the clinical leadership team observes them in their first through fourth sessions randomly to see live examples of their diagnostic skills, provision of psychoeducation, and proficiency in the development of ERP hierarchies. The full-time therapists attend 2 hours per week of clinical supervision or case consultation as well as a 3-, 6-, 9-, and 12-month clinical advising review of their cases.

### Assessments

Assessments were emailed to patients as links and were conducted at the initial diagnostic assessment, at treatment midpoint (after 6 twice-weekly therapy sessions), and the endpoint (after 6 weekly 30-minute check-in sessions). The use of patient-rated scales as the outcome variables of interest reduced the risk of therapist bias that may occur with clinician-rated scales. Follow-up assessments were sent to patients at the therapy visit closest in time to 3, 6, 9, and 12 months after their endpoint assessment. The majority of these follow-up sessions were 30-minute brief check-in sessions, as most had transitioned to less frequent visits (30-minute check-ins twice monthly to once every 3 months).

Dimensional Obsessive-Compulsive Scale (DOCS) [23] is a 20-item self-report measure of OCD symptom severity across four domains: contamination, responsibility for harm or mistakes, unacceptable thoughts, and incompleteness or symmetry. The DOCS has shown good psychometric properties, including strong convergent validity with the Yale-Brown Obsessive Compulsive Scale (*r*=0.54) and the Obsessive-Compulsive Inventory—Revised (*r*=0.69), and is sensitive to the effects of treatment.

The DIAMOND severity scale [21] is a 2-item clinician-rated assessment of the overall severity of an individual’s emotional distress and functional impairment related to OCD symptoms. The clinician makes separate ratings of an individual’s emotional distress and functional impairment on a scale ranging from 1 (Normal) to 7 (Extreme), and the higher of the two ratings is taken as the total severity score.

Depression, Anxiety, and Stress Scales (DASS-21) [24] is a 21-item self-report measure of symptoms of depression, anxiety, and stress. It has been widely used in previous research and has consistently shown good psychometric qualities.

Quality of Life Enjoyment and Satisfaction Questionnaire—Short Form [25] is a 14-item self-report assessment of quality of life across a variety of life domains. It has demonstrated good psychometric properties in previous research.

### Statistical Analyses

All data were deidentified prior to analysis. We analyzed data for those patients who completed at least the initial and the endpoint outcome assessments for the DOCS, the primary outcome measure. The majority also had a midpoint assessment. Data analysis was conducted using a linear mixed model (in part to handle missing data) with assessment time point as a fixed factor, patient as a random factor, and DOCS as the primary dependent variable. Secondary outcome analyses for the DASS-21 subscales of depression, anxiety, and stress, and the Quality of Life Enjoyment and Satisfaction Questionnaire—Short Form, were analyzed using the same model. A tertiary outcome was follow-up symptom severity ratings on the DOCS at 3, 6, 9, and 12 months from the endpoint assessment; this was also conducted using linear mixed models with assessment time point as a fixed factor (initial, 3-month, 6-month, 9-month, and 12-month time points), patient as a random factor, and DOCS as the primary dependent variable. Statistical significance was determined using an alpha of .05. Outcome analyses were conducted using SPSS version 27.0.0.0 (IBM Corp). We calculated Hedges g effect sizes using R (R Foundation for Statistical Computing).

### Ethical Considerations

The analysis conducted in this study did not require research ethics board review as it does not meet the criteria for Human Subject Research as defined by federal regulations for human subject protections, 45 CFR 46.102(e); this is a secondary analysis of de-identified data from clinical records, obtained and analyzed retrospectively, and was not the result of a research intervention or interaction.

NOCD’s Privacy Policy complies with the UK Data Protection Act of 2018, as well as the European Union’s General Data Protection Regulation privacy law. All patients who are treated by NOCD must accept NOCD’s Privacy Policy, which discusses how personal data are used, by whom, and for what purpose.

## Results

### Sample

We analyzed data collected from patients who started treatment between January 1, 2020, and June 30, 2021. (It is important to note that this date range was chosen to capture outcomes from when NOCD started enrolling substantial numbers of patients until the treatment protocol introduced minor changes in late August 2021; individuals who started as late as June 30, 2021, for example, would have finished treatment before these came into effect.)

**Figure 1:**
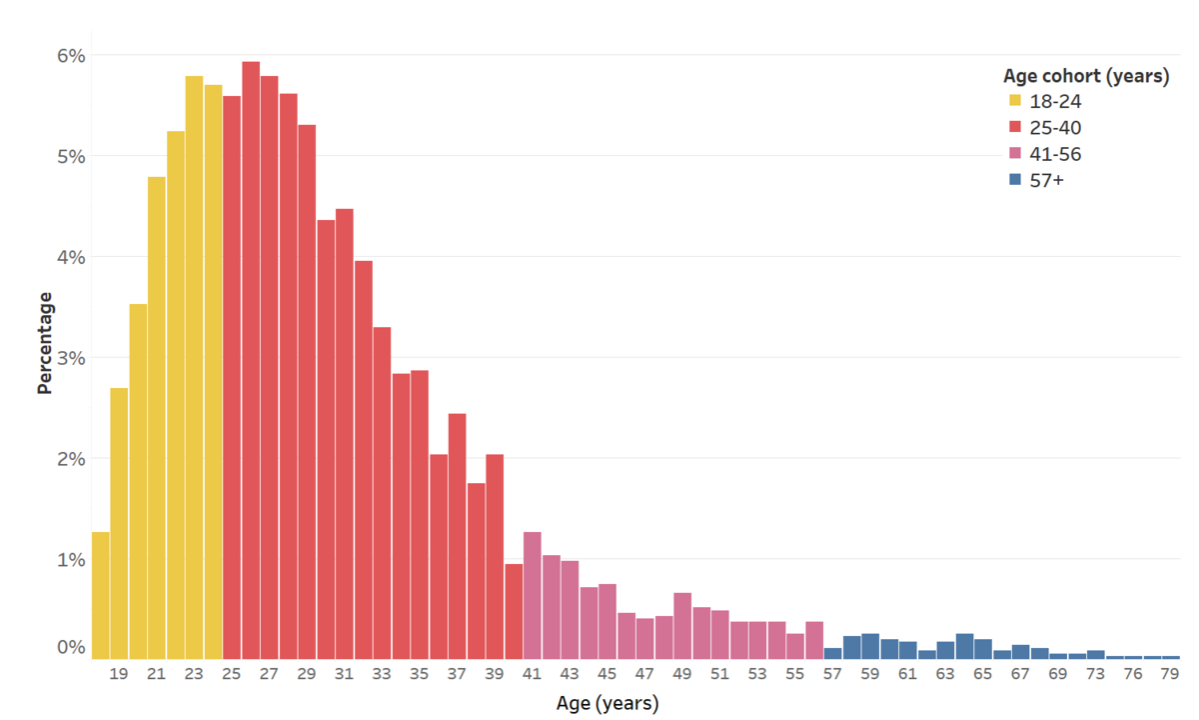
Age distribution.

We analyzed data from adults (aged ≥18 years) with a diagnosis of OCD who had at least an initial and endpoint assessment with the primary outcome measure, the DOCS. Data from 3552 patients who met these criteria were analyzed. Those who had fewer than 5 sessions were excluded (representing <0.1% of the sample), as this indicated that the treatment was likely interrupted, and outcomes were not available. The mean age was 29.9 (SD 9.3) years, range 18-79 years (Figure 1). In terms of gender, 55.88% (1985/3552) identified as female and 37.56% (1334/3552) identified as male (6.56% [233/3552] indicated nonbinary or another gender-expansive identity or did not provide this information). Regarding comorbidities, 36.4% (1293/3552) had a comorbid anxiety disorder, 32.8% (1165/3552) had a comorbid mood disorder, 10.3% (366/3552) had a comorbid OCD-related disorder, 5.3% (188/3552) had a trauma and stress-related disorder (posttraumatic stress disorder or acute stress disorder), 1.8% (64/3552) had a substance use disorder, 11.2% (398/3552) had another comorbid disorder, and 62% (2202/3552) had no comorbid disorders (Table S3 in Multimedia Appendix 1).

### NOCD App Use, Messaging, and NOCD Web-Based Community Posts

The app was used by 3529/3552 (99.4%) of patients at least once, and 3515/3552 (99%) sent at least one text message. Further, 1932/3552 (45.6%) made at least one community post. The mean number of app usages was 454.7 (SD 852.8), and the mean number of community posts was 55.8 (SD 282.0).

### Treatment Duration

The mean treatment duration was 11.54 (SD 3.96) weeks (median=10.71, mode=9), the mean number of therapist sessions was 13.0 (SD 1.3; median=13.0, mode=13), and the mean number of therapist hours was 10.6 (SD 1.1; median=10.5, mode=10.5). Of the total 3552 sample, 53% (n=1883) had >14 sessions before the 3-month follow-up; of these, the mean number of 60-minute sessions was 7.7 (SD 2.0; the mean for those with 13 sessions was 7.0, SD 1.0) and the mean number of 30-minute check-in sessions was 9.1 (SD 1.7; the mean for those with 13 sessions was 5.8, SD 0.9). This amounts to a mean total of 16.8 (SD 2.2) sessions in those with >14 sessions; the majority of the additional sessions, if they were conducted, were check-in sessions.

### OCD Symptom Results

**Figure 2:**
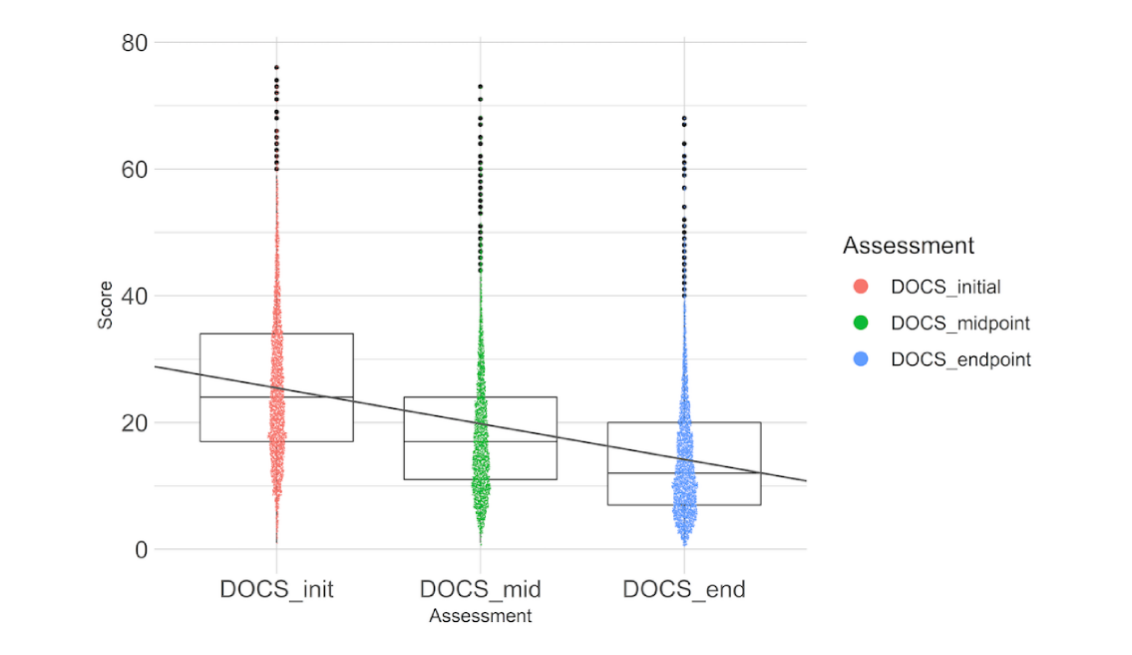
Changes in obsessive-compulsive disorder symptoms as assessed by the Dimensional Obsessive-Compulsive Scale (DOCS) with treatment (*P*<.001 for DOCS_mid compared with initial scores and *P*<.001 for DOCS_end compared with initial scores).

NOCD treatment resulted in a significant decrease in patient-rated OCD symptoms (DOCS scores; F_6646.02_=2810.08, *P*<.001; initial to endpoint Hedges g=1.0: “large” effect size). On the total sample level, DOCS scores improved from a mean of 26.0 (SD 12.3) to a mean of 14.7 (SD 9.8), representing a mean 11.3-point decrease (43.4%). On the individual patient level, the median DOCS score improvement was 45%. Note that we report the median for the individual score change rather than the mean, as it is a better representation of the central tendency for percentage change for these data. This is due to the fact that individuals’ scores can worsen more than 100% but cannot improve more than 100%, which can result in a skewed distribution (Figure 2).

By midpoint, there were also statistically significant improvements; DOCS scores improved to a mean of 18.6 (SD 10.6), representing a mean 7.3-point decrease (28.2%). On the individual patient level, median DOCS score improvement was 30.8%.

Further, 62.9% (2234/3552) met the criteria as full “responders,” defined as a ≥35% reduction in OCD symptoms [26]. A total of 74.2% (2636/3552) met the criteria as achieving either partial (25%-35% reduction) or full response.

### Follow-up Scores at 3, 6, 9, and 12 Months

**Figure 3:**
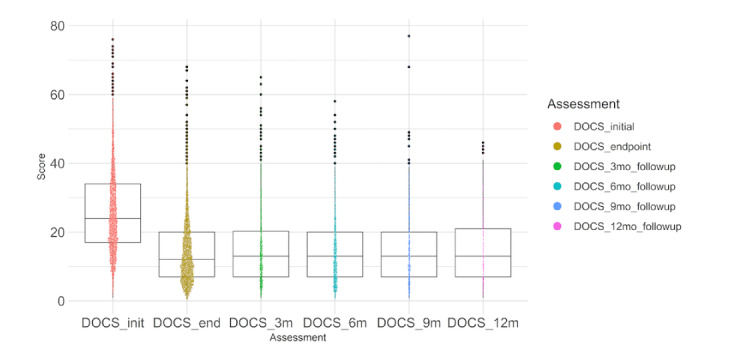
Longitudinal follow-up of obsessive-compulsive disorder symptoms as assessed by the Dimensional Obsessive-Compulsive Scale (DOCS; *P*<.001 for DOCS_end, DOCS_3m, DOCS_6m, DOCS_9m, and DOCS_12m compared with initial scores).

Of the whole 3552 sample, 1633 (46%) did a 3-, 6-, 9-, or 12-month follow-up. At 3, 6, 9, and 12 months post the endpoint assessment, most patients had maintained their improvements in all symptom and quality of life domains. This was evidenced by mean DOCS, DASS depression, DASS anxiety, DASS stress, and QLESQ-SF scores at 3, 6, 9, and 12 months, which were similar to scores at the endpoint of treatment and remained significantly different from the initial assessment (Figure 3 and Tables S1 and S2 in Multimedia Appendix 1).

### Depression, Anxiety, Stress, and Quality of Life Results

**Figure 4:**
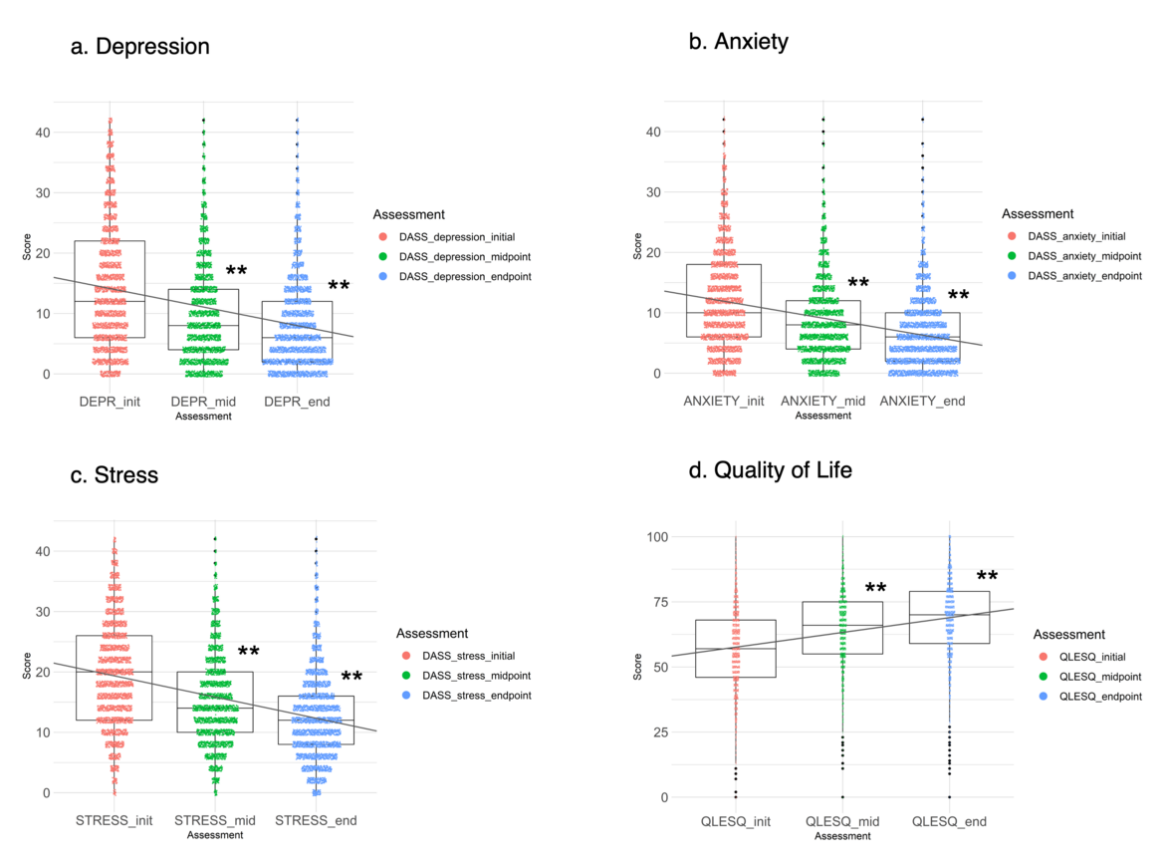
Changes in depression, anxiety, stress, and quality of life with treatment. DASS: Depression, Anxiety, and Stress Scales; DEPR: depression; QLESQ: Quality of Life Enjoyment and Satisfaction Questionnaire; **P<.001 compared with initial scores.

Treatment resulted in significant improvements on the DASS depression (F_6647.79_=972.91, *P*<.001; initial to endpoint Hedges g=0.66), DASS anxiety (F_6659.83_=1162.76, *P*<.001; initial to endpoint Hedges g=0.76), DASS stress (F_6645.12_=1387.22, *P*<.001; initial to endpoint Hedges g=0.87), and the QLESQ-SF (F_6156.13_=1140.66, *P*<.001; initial to endpoint Hedges g=0.76) (Figure 4 and Tables 1 and 2).

**Table 1:** Clinical assessments by treatment time point.

Outcome scale and assessment time point		Valid, n	Missing, n	Mean	SD	Mean 95.0% CI, lower bound	Mean 95.0% CI, upper bound	Median	Median 95.0% CI, lower bound	Median 95.0% CI, upper bound
**DOCS^a^**										
	Initial	3552	0	26.0	12.3	25.5	26.4	24	24	25
	Midpoint	3037	515	18.6	10.6	18.2	19.0	17	17	18
	Endpoint	3552	0	14.7	9.8	14.4	15.0	12	12	13
**DASS^b^ depression**										
	Initial	3551	1	14.5	10.4	14.1	14.8	12	12	14
	Midpoint	3032	520	10.2	8.7	9.9	10.5	8	8	10
	Endpoint	3526	26	8.4	7.9	8.1	8.6	6	6	8
**DASS anxiety**										
	Initial	3551	1	12.1	8.3	11.8	12.4	10	10	12
	Midpoint	3033	519	8.6	6.7	8.4	8.8	8	8	10
	Endpoint	3528	24	6.5	5.9	6.3	6.7	6	6	8
**DASS stress**										
	Initial	3550	2	19.7	8.8	19.	19.9	20	20	22
	Midpoint	3033	519	15.1	7.7	14.9	15.4	14	14	16
	Endpoint	3528	24	12.6	7.3	12.4	12.9	12	12	14
**QLESQ^c^**										
	Initial	3469	83	57.1	16.2	56.6	57.7	57	57	59
	Midpoint	2764	788	64.5	14.9	63.9	65.0	66	66	68
	Endpoint	3295	257	68.4	15.1	67.9	69.0	70	70	71

^a^DOCS: Dimensional Obsessive-Compulsive Scale.

^b^DASS: Depression Anxiety and Stress Scale—21.

^c^QLESQ: Quality of Life Enjoyment and Satisfaction Questionnaire—Short Form.

**Table 2:** Changes in OCD,^a^ depression, anxiety, and stress symptoms and quality of life by assessment time point.

Outcome scale and assessment			Score change		SE		Score change 95% CI, lower bound		Score change 95% CI, upper bound		Percentage change		*df*		*t*		Sig.^b^		Hedges g effect size		Hedges g 95% CI, lower bound		Hedges g 95% CI, upper bound
**DOCS^c^**																							
	Midpoint	–7.4		0.2		–7.7		–7.0		–28.4		6680.86		–45.71		<.001		0.66		0.63		0.69	
	Endpoint	–11.3		0.1		–11.6		–11.0		–43.4		6583.44		–73.97		<.001		1.00		0.93		1.03	
**DASS^d^ depression**																							
	Midpoint	–4.4		0.1		–4.7		–4.1		–30.4		6696.36		–29.16		<.001		0.48		0.45		0.51	
	Endpoint	–6.2		0.1		–6.4		–5.9		–42.5		6571.51		–42.96		<.001		0.66		0.62		0.69	
**DASS anxiety**																							
	Midpoint	–3.6		0.1		–3.9		–3.4		–29.9		6715.28		–29.11		<.001		0.50		0.46		0.53	
	Endpoint	–5.6		0.1		–5.9		–5.4		–46.4		6572.55		–47.65		<.001		0.76		0.72		0.80	
**DASS stress**																							
	Midpoint	–4.6		0.1		–4.9		–4.3		–23.4		6702.45		–32.23		<.001		0.59		0.55		0.63	
	Endpoint	–7.0		0.1		–7.3		–6.8		–35.8		6554.35		–51.96		<.001		0.87		0.83		0.91	
**QLESQ^e^**																							
	Midpoint	7.7		0.3		7.2		8.2		13.5		6227.25		29.47		<.001		0.55		0.52		0.59	
	Endpoint	11.6		0.2		11.1		12.1		20.3		6107.25		46.98		<.001		0.76		0.72		0.79	

^a^OCD: obsessive-compulsive disorder.

^b^Sig.: significance probability.

^c^DOCS: Dimensional Obsessive-Compulsive Scale.

^d^DASS: Depression Anxiety and Stress Scale–21.

^e^QLESQ: Quality of Life Enjoyment and Satisfaction Questionnaire—Short Form.

### Post Hoc Analysis of Outcomes Stratified by Starting Clinician-Rated Severity Level

To determine how treatment response differed by different initial severity levels of OCD, we used the DIAMOND scale at the initial assessment to stratify patients into three groups of severity ratings: “Mild” (severity score of 2 or 3), “Moderate” (severity score of 4 or 5), or “Severe” (severity score of 6 or 7). Moreover, of the 3552 patients, 596 (17%) were missing DIAMOND severity scale scores. Of these, there was a median 46.86% reduction in DOCS scores and a 64.3% response rate. For DOCS scores, on the individual patient level, the Mild group (n=679, 19%) had a median 50.0% reduction, the Moderate group (n=2079, 59%) a median 42.9% reduction, and the Severe group (n=198, 6%) a median 44.6% reduction. Response rates from the DOCS were 68.8% for Mild, 60.7% for Moderate, and 61.6% for Severe.

## Discussion

Patients with OCD treated with digital teletherapy using ERP show significant improvement in symptoms. OCD symptoms were reduced by 43.4%. Moreover, 62.9% (2234/3552) were classified as full responders, and 74.2% (2636/3552) had partial or full response. Treatment also resulted in improvements in the common comorbid symptoms of depression, anxiety, and stress and resulted in a significant improvement in quality of life. This provides evidence that a single, focused OCD treatment can result in an overall reduction of multiple disabling and distressing symptoms and improve the lives of patients. This is notable considering the fact that OCD is a chronic illness that individuals on average have for 11 years before receiving treatment [27]. Long-term follow-up data at 3-, 6-, 9-, and 12 months post treatment showed overall maintenance of gains from the initial treatment period.

These results demonstrate not only the magnitude of the effect of this treatment model on OCD and comorbid symptoms but also its efficiency in terms of cost and time savings. The time frame of these improvements was less than 12 weeks and less than 11 total therapist hours, on average. This is less than half the total therapist time and less than half of the duration of traditional once-weekly outpatient ERP [12]. This has the potential for substantial cost savings for patients and third-party payors such as health insurers.

This treatment format was inspired by a treatment previously developed and tested [17] to provide evidence-based ERP treatment for OCD, in a manner that is efficient in terms of total therapist time. The OCD symptom reduction results in the current NOCD-treated sample are similar to those achieved in that study. Yet, direct comparisons are limited by the fact that the current sample was from a “real-world” clinical setting rather than a controlled research setting with more selective inclusion and exclusion criteria. Other differences that preclude direct comparisons include, but are not limited to, the fact that NOCD used the patient-rated DOCS scale, whereas the previous study used the clinician-rated Yale-Brown Obsessive-Compulsive Scale [28] as the primary outcome measure. Further, NOCD treatment consisted of face-to-face teletherapy rather than in-person therapy.

There are other important additional elements of treatment in the NOCD model that impact patient experience and may have influenced outcomes. Additional support for patients was available between sessions through patient-therapist SMS messaging. Patients also had 24-hour access to NOCD’s web-based support community, consisting of messaging boards from others with OCD and organized around common OCD subtypes. This allows people to find others who experience similar symptoms, which can help reduce the sense that their OCD symptoms are a rare or unique type of OCD and therefore difficult to treat or may not even be OCD. This can be an important experience for those with OCD, given the broad and heterogeneous content of obsessional thoughts [29]. In addition, only a limited number of subtypes such as those involving contamination or washing, checking symptoms, and ordering or symmetry are typically described in the literature and are widely known, so certain OCD symptoms might be missed or misidentified by clinicians, family members, and patients themselves. In addition, patients had peer support from former patients who had completed NOCD treatment. When used, this would occur in the interval between contacting the initial call center for NOCD and their first diagnostic appointment with their therapist. The peer support may encourage people to follow through with scheduling and attending their first assessment meeting and beginning treatment. This additional support may be particularly useful due to the fact that ERP can be challenging for individuals to engage in; this is because, by necessity, ERP’s therapeutic mechanisms are predicated on inducing distress (exposures) and eliminating behaviors that temporarily relieve distress (response prevention) but that perpetuate the cycle of obsessions and compulsions. Future investigations will quantify if, and to what degree, these additional digital and personal treatment elements affected clinical outcomes and patient experience.

Technology assistance likely played an important role in this treatment’s ability to both engage and treat a large number of patients in wide-ranging geographic locations and to achieve a high mean rate of symptom improvement and a high rate of treatment response. Teletherapy using video allows people in remote locations to access treatment and to be able to complete, in-session, in vivo exercises in places and situations that are most relevant to, or triggering of, their symptoms. Previous studies of remote therapy demonstrate effect sizes that are similar to controlled studies of in-person treatment (see the meta-analysis [13]). The effect size for OCD symptom severity reduction in the current analysis of g=1.0 (“large” effect size) (95% CI .93 to 1.03) is similar to that found in a recent meta-analysis of controlled studies of ERP vs psychological placebo (g=1.13, 95% CI 0.71 to 1.55; 10 studies) [5]. Importantly, the current results are observed in a cohort that is one to two orders of magnitude larger than previous controlled ERP studies [5], providing strong evidence that virtual face-to-face ERP can be at least as effective as in-person ERP.

While most previous studies of ERP *efficacy* have come from clinical research trials, a meta-analysis of *effectiveness* studies of CBT in real-world clinical settings found an effect size for reduction of OCD symptoms across 11 studies of *d*=1.32 (95% CI: 1.19 to 1.45) [7]. A study published more recently examined CBT outcomes in an outpatient setting and found a mean 47.09% reduction in OCD symptoms on the Obsessive-Compulsive Inventory—Revised (n=451 at baseline and n=235 post treatment, effect size *d*=1.18) [30]. However, some differences limit direct comparisons to the current results because other studies used different OCD outcome rating scales (primarily the clinician-rated Yale-Brown Obsessive-Compulsive Scale or the patient-rated Obsessive-Compulsive Inventory—Revised rather than the DOCS) and had much smaller sample sizes.

Aside from video teletherapy, there are other technology-based features of this treatment that may have enhanced patient engagement. This includes integrated SMS messaging that allowed for increased continuity of treatment; patients could obtain advice and assistance when doing homework assignments in between sessions or when encountering unexpected situations that lead to obsessions and distress. This both helps keep treatment momentum and helps patients feel a more continuous sense of support. Further, the NOCD app has built-in tools for creating ERP hierarchies for exposure treatment planning. In addition, there are tools such as distress ratings to track progress during exposures and to track exposure-to-exposure progress, all of which can be visualized graphically by the patients and therapists. In this sample, almost all used the NOCD app and almost half made at least one post in the online community, with an average of approximately 56 posts per person. The specific effects of these technology features, as well as the effects of peer support and online community support, can be measured and evaluated in future analyses.

Another finding of note in this analysis was that symptom improvements were relatively similar for those with mild, moderate, and severe OCD symptoms. Overall mean symptom improvements were thus not driven only by those, for example, on the milder end of the symptom severity spectrum. Rather, the treatment model works well for those with a wide range of baseline symptoms, including those with severe OCD.

There are several limitations of this analysis, which are mostly due to its observational nature. Data were missing for some patients for certain rating scales (Table 1). Although all therapists received training in conducting ERP from NOCD’s curriculum and learned the overall structure of the treatment model, therapy sessions were not videotaped to ensure treatment fidelity and consistency from therapist to therapist, as in a research study. However, therapists were regularly audited in terms of outcomes, patient feedback, and patient retention and were assisted in improving in any of these areas if necessary. Another limitation to the generalizability of the results is that the treatment model allowed for some flexibility; for example, therapists sometimes extended treatment beyond the 3 weeks of twice-weekly therapy or 6 weeks of once-weekly brief therapy check-ins if they deemed it important for patient improvement. Another limitation is the use of the DOCS as the primary OCD outcome measure. As a patient-rated measure, it depends on patients’ understanding of their symptoms in the framework of OCD. This could be problematic if patients do not recognize that some of their experiences are OCD symptoms, or if they believe that some experiences are OCD symptoms when they are not, which can result in erroneously low or high scores, respectively. This is a limitation, however, of all OCD rating scales to varying degrees. In addition, the majority of those whom therapists determined in the initial diagnostic assessment to have “extreme” OCD symptoms on the DIAMOND severity scale (aside from n=16 for whom exceptions were made on a case-by-case basis) were not treated and were instead referred to a higher level of care. Thus, although outcomes were similar for those with mild to severe cases, there is less certainty about generalization to those with extreme OCD severity. Another limitation is that 3-, 6-, 9-, and 12-month follow-up data were not available for many who completed the treatment. Thus, it remains unknown whether the proportions of those for whom 3-, 6-, 9-, and 12-months follow-up data were not provided represented individuals who were doing worse and sought other treatment, or were doing much better and did not see the need to continue these sessions. Further, even for those who provided data during this follow-up period, some may have engaged in other concurrent treatments.

In sum, ERP delivered in a technology-assisted video teletherapy treatment format results in clinically significant symptom and quality of life improvements in a real-world sample, on a large scale. This can provide a readily accessible means of obtaining effective, evidence-based treatment of OCD. Further, the relatively efficient treatment that is delivered can represent substantial cost savings for patients and third-party payors over traditional weekly outpatient face-to-face ERP.

## Appendix

Multimedia Appendix 1 Supplemental document that contains visualizations of analysis pertaining to characterization of the data in terms of demographics and tables.

## Abbreviations

CBT: cognitive-behavioral therapy
DASS: Depression, Anxiety, and Stress Scales
DIAMOND: Diagnostic Interview for Anxiety, Mood, and OCD and Related Neuropsychiatric Disorders
DOCS: Dimensional Obsessive-Compulsive Scale
ERP: exposure and response prevention
OCD: obsessive-compulsive disorder
