# Supplementary material for: Online Video Teletherapy Treatment of Obsessive-Compulsive Disorder Using Exposure and Response Prevention: Clinical Outcomes From a Retrospective Longitudinal Observational Study
Source: J Med Internet Res. 2022 May 19;24(5):e36431. doi: 10.2196/36431 (PMC9164091; doi:10.2196/36431)

Supplemental Materials for

**Online Video Teletherapy Treatment of Obsessive-Compulsive Disorder Using Exposure and Response Prevention: Clinical Outcomes from a Retrospective Longitudinal Observational Study**

Jamie D. Feusner^1-5^, Nicholas R. Farrell^1^, Jeremy Kreyling^1^, Patrick B. McGrath^1^, Andreas Rhode^1^, Ted Faneuff^1^, Stephanie Lonsway^1^, Reza Mohideen^1^, John Jurich^1^, Larry Trusky^1^, Stephen M. Smith^1^

**Author Affiliations:**

^1^NOCD, Inc., Chicago, IL, 60611, USA

^2^Department of Psychiatry, University of Toronto, Toronto, ON, Canada

^3^Centre for Addiction and Mental Health, Toronto, ON, Canada

^4^Department of Psychiatry and Biobehavioral Sciences, University of California Los Angeles, Los Angeles, CA, USA

^5^Department of Women’s and Children’s Health, Karolinska Institutet, Stockholm, Sweden

Table of Contents

Figure S1……………………………………………………………………………………. Page 2

Table S1…………………………………………………..………………………………… Page 3

Table S2…………………………………………………..………………………………… Page 5

Table S3…………………………………………………..………………………………… Page 7

Fig. S1. Geographical distribution of participants


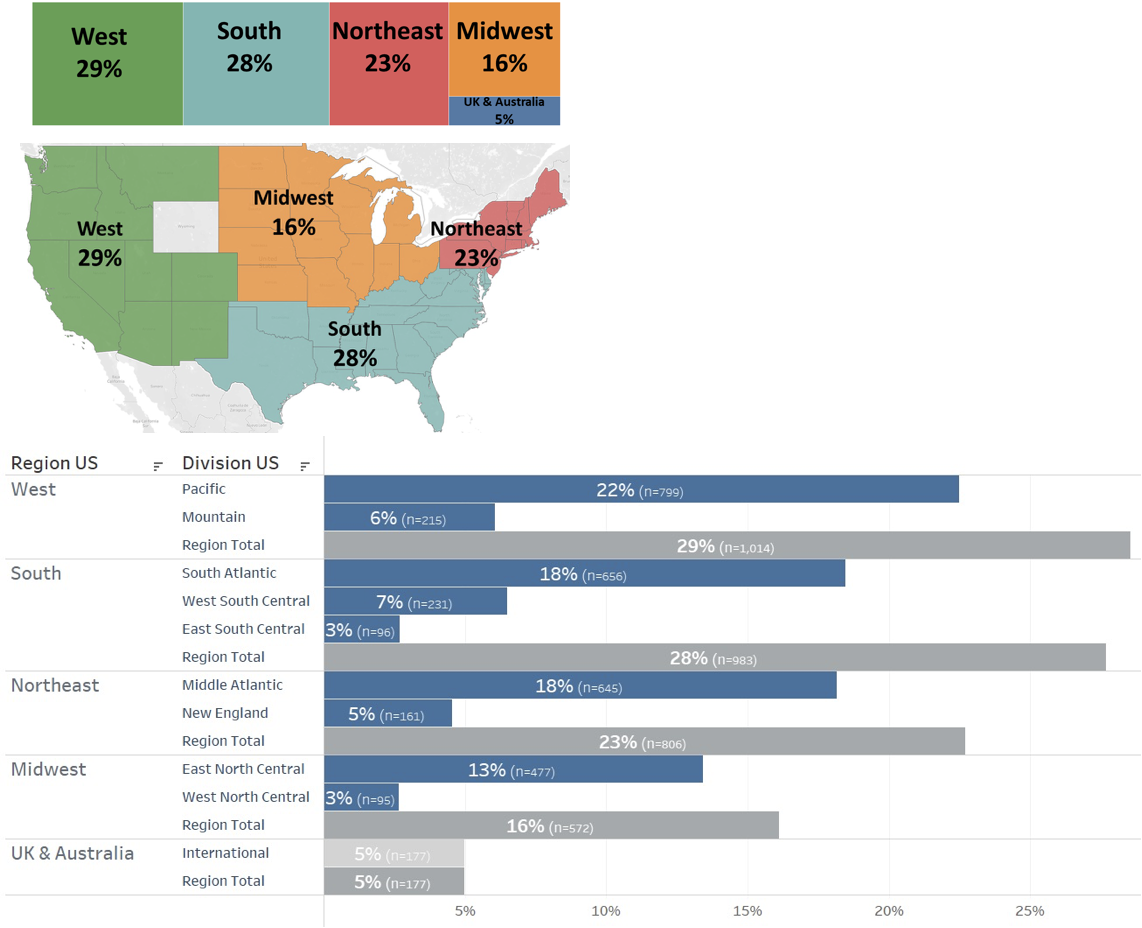


Table S1

|  |  | Valid N | Missing | Mean | STD | 95.0% Lower CL for Mean | 95.0% Upper CL for Mean | Median | 95.0% Lower CL for Median | 95.0% Upper CL for Median |
| --- | --- | --- | --- | --- | --- | --- | --- | --- | --- | --- |
| DOCS | initial | 3552 | 0 | 26 | 12 | 26 | 26 | 24 | 24 | 25 |
|  | 3 mo | 820 | 2732 | 15 | 11 | 15 | 16 | 13 | 13 | 14 |
|  | 6 mo | 1068 | 2484 | 15 | 10 | 14 | 15 | 13 | 13 | 14 |
|  | 9 mo | 533 | 3019 | 15 | 10 | 14 | 16 | 13 | 13 | 15 |
|  | 12 mo | 249 | 3303 | 15 | 10 | 14 | 16 | 13 | 11 | 16 |
| DASS_depression | initial | 3551 | 1 | 14 | 10 | 14 | 15 | 12 | 12 | 14 |
|  | 3 mo | 803 | 2749 | 9 | 9 | 9 | 10 | 6 | 6 | 8 |
|  | 6 mo | 1052 | 2500 | 8 | 8 | 8 | 9 | 6 | 6 | 8 |
|  | 9 mo | 524 | 3028 | 8 | 8 | 7 | 9 | 6 | 6 | 8 |
|  | 12 mo | 244 | 3308 | 9 | 9 | 7 | 10 | 6 | 6 | 8 |
| DASS_anxiety | initial | 3551 | 1 | 12 | 8 | 12 | 12 | 10 | 10 | 12 |
|  | 3 mo | 803 | 2749 | 7 | 7 | 7 | 8 | 6 | 6 | 8 |
|  | 6 mo | 1052 | 2500 | 7 | 6 | 6 | 7 | 6 | 6 | 8 |
|  | 9 mo | 524 | 3028 | 7 | 6 | 6 | 7 | 6 | 6 | 8 |
|  | 12 mo | 244 | 3308 | 7 | 6 | 6 | 7 | 4 | 4 | 6 |
| DASS_stress | initial | 3550 | 2 | 20 | 9 | 19 | 20 | 20 | 20 | 22 |
|  | 3 mo | 803 | 2749 | 13 | 8 | 13 | 14 | 12 | 12 | 14 |
|  | 6 mo | 1052 | 2500 | 13 | 8 | 12 | 13 | 12 | 12 | 14 |
|  | 9 mo | 524 | 3028 | 13 | 8 | 13 | 14 | 12 | 12 | 14 |
|  | 12 mo | 244 | 3308 | 13 | 8 | 12 | 14 | 12 | 12 | 14 |
| QLESQ | initial | 3469 | 83 | 57 | 16 | 57 | 58 | 57 | 57 | 59 |
|  | 3 mo | 981 | 2571 | 67 | 16 | 66 | 68 | 68 | 68 | 70 |
|  | 6 mo | 897 | 2655 | 67 | 16 | 66 | 68 | 70 | 70 | 71 |
|  | 9 mo | 476 | 3076 | 68 | 15 | 67 | 69 | 68 | 68 | 71 |
|  | 12 mo | 205 | 3347 | 66 | 15 | 64 | 68 | 68 | 66 | 71 |

Table S2

| **Outcome scale** | **Assessment timepoint** | **Score change** | **Std. Error** | **Score change 95% CI Lower Bound** | **Score change 95% CI Upper Bound** | **% change** | **df** | **t** | **Sig.** | **Hedges g effect size** | **Hedges g 95% CI Lower Bound** | **Hedges g 95% CI Upper Bound** |
| --- | --- | --- | --- | --- | --- | --- | --- | --- | --- | --- | --- | --- |
|  |  |  |  |  |  |  |  |  |  |  |  |  |
| **DOCS** | 3 mo | -10.89 | 0.32 | -11.51 | -10.27 | -41.95 | 3250.05 | -34.40 | <.001 | 0.93 | 0.86 | 1.01 |
|  | 6 mo | -11.62 | 0.28 | -12.18 | -11.07 | -44.77 | 3234.29 | -41.18 | <.001 | 1.07 | 1.00 | 1.14 |
|  | 9 mo | -11.73 | 0.38 | -12.47 | -10.98 | -45.17 | 3188.41 | -30.76 | <.001 | 1.05 | 0.94 | 1.16 |
|  | 12 mo | -11.33 | 0.54 | -12.39 | -10.27 | -43.64 | 3136.50 | -20.97 | <.001 | 1.00 | 0.83 | 1.16 |
| **DASS_depression** | 3 mo | -5.45 | 0.29 | -6.02 | -4.88 | -37.60 | 3328.41 | -18.84 | <.001 | 0.57 | 0.50 | 0.65 |
|  | 6 mo | -5.82 | 0.26 | -6.32 | -5.32 | -40.16 | 3300.94 | -22.64 | <.001 | 0.61 | 0.54 | 0.67 |
|  | 9 mo | -6.29 | 0.35 | -6.97 | -5.61 | -43.41 | 3264.18 | -18.08 | <.001 | 0.66 | 0.55 | 0.76 |
|  | 12 mo | -6.08 | 0.49 | -7.05 | -5.11 | -41.96 | 3218.03 | -12.29 | <.001 | 0.63 | 0.48 | 0.77 |
| **DASS_anxiety** | 3 mo | -4.72 | 0.23 | -5.18 | -4.26 | -38.96 | 3325.62 | -20.19 | <.001 | 0.60 | 0.53 | 0.68 |
|  | 6 mo | -5.38 | 0.21 | -5.78 | -4.97 | -44.35 | 3291.28 | -25.84 | <.001 | 0.72 | 0.65 | 0.80 |
|  | 9 mo | -5.43 | 0.28 | -5.99 | -4.88 | -44.84 | 3261.78 | -19.30 | <.001 | 0.73 | 0.63 | 0.83 |
|  | 12 mo | -5.96 | 0.40 | -6.74 | -5.17 | -49.17 | 3216.49 | -14.88 | <.001 | 0.82 | 0.67 | 0.98 |
| **DASS_stress** | 3 mo | -6.13 | 0.27 | -6.65 | -5.61 | -31.20 | 3581.81 | -23.11 | <.001 | 0.72 | 0.64 | 0.80 |
|  | 6 mo | -6.62 | 0.24 | -7.08 | -6.16 | -33.67 | 3539.67 | -28.04 | <.001 | 0.79 | 0.72 | 0.87 |
|  | 9 mo | -6.40 | 0.32 | -7.02 | -5.77 | -32.54 | 3521.16 | -20.02 | <.001 | 0.77 | 0.66 | 0.87 |
|  | 12 mo | -6.88 | 0.45 | -7.77 | -5.98 | -34.97 | 3478.82 | -15.12 | <.001 | 0.82 | 0.68 | 0.96 |
| **QLESQ** | 3 mo | 10.22 | 0.44 | 9.37 | 11.07 | 17.89 | 3334.74 | 23.47 | <.001 | 0.68 | 0.61 | 0.74 |
|  | 6 mo | 10.29 | 0.45 | 9.41 | 11.18 | 18.02 | 3332.71 | 22.75 | <.001 | 0.67 | 0.60 | 0.74 |
|  | 9 mo | 10.32 | 0.60 | 9.15 | 11.49 | 18.06 | 3298.85 | 17.27 | <.001 | 0.59 | 0.48 | 0.69 |
|  | 12 mo | 9.07 | 0.88 | 7.34 | 10.80 | 15.87 | 3256.19 | 10.26 | <.001 | 0.45 | 0.28 | 0.63 |

Table S3. Psychiatric comorbidity of the sample


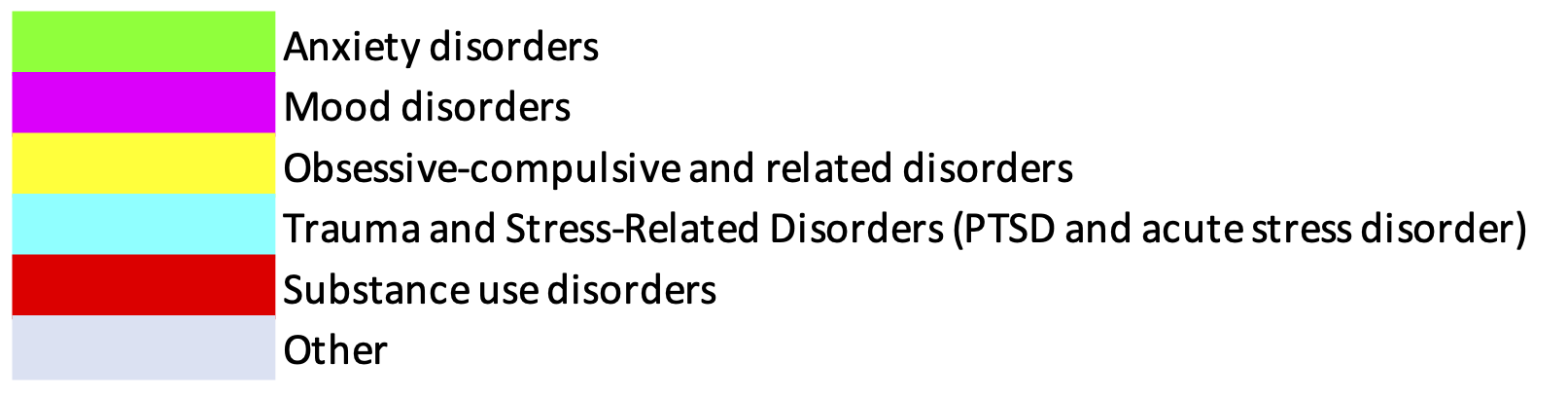


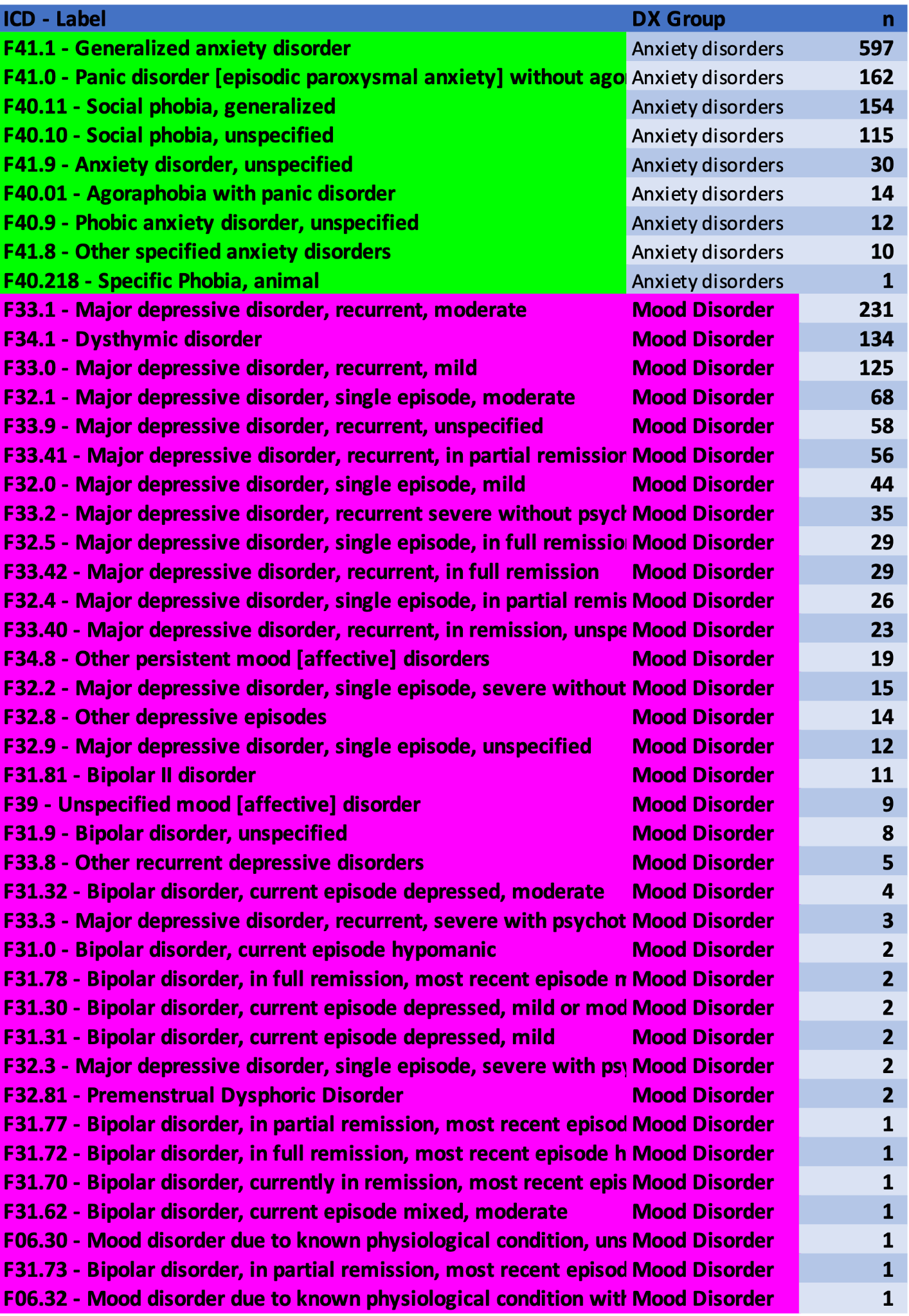


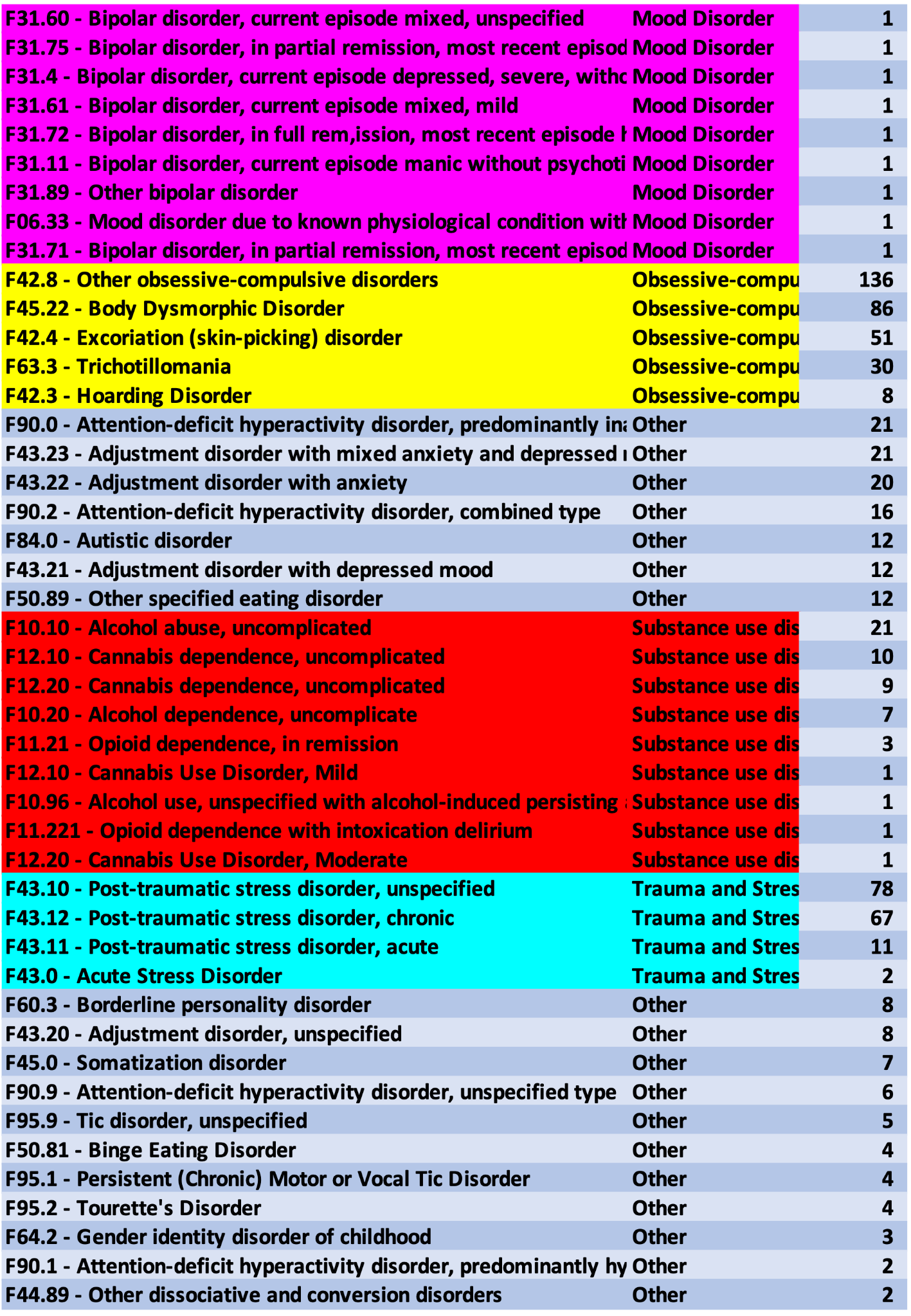

Supplement: Multimedia Appendix 1 [file jmir_v24i5e36431_app1.docx]
